# A pilot prospective study of arterial stiffness during weight restoration in adolescents with anorexia nervosa

**DOI:** 10.1007/s40519-025-01793-6

**Published:** 2025-11-18

**Authors:** Elizabeth Y.F. Tee, Simon D. Clarke, Linette Gomes, Basiliki Lampropoulos, Gail Anderson, Christine Wearne, Aravinda Thiagalingam, Afraz Zaman, Michael R. Kohn

**Affiliations:** 1 Sydney Medical School, Faculty of Medicine and Health, University of Sydney, Sydney, Australia; 2 Department of Adolescent and Young Adult Medicine, Westmead Hospital, Sydney, Australia; 3 Department of Cardiology, Westmead Hospital, Sydney, Australia; 4 Department of Child and Adolescent Psychiatry, Westmead Hospital, Sydney, Australia; 5 Centre for Research into Adolescent’s Health (CRASH), Sydney, Australia; 6 University of Western Sydney, School of Medicine, Sydney, Australia

**Keywords:** Arterial stiffness, Anorexia nervosa, Adolescents, Cardiovascular

## Abstract

**Purpose:**

Carotid–femoral pulse wave velocity (cfPWV), an index of arterial stiffness, is one of the earliest indicators of cardiovascular risk. Studies of adolescents with anorexia nervosa have demonstrated increased arterial stiffness compared to healthy controls. Little information is available on the effect of weight restoration on arterial stiffness in adolescents with anorexia nervosa.

**Methods:**

This pilot longitudinal study examined changes in arterial stiffness during weight restoration in adolescent females admitted to an inpatient eating disorder unit. Female adolescents aged 15–19 years with a diagnosis of anorexia nervosa and a body mass index (BMI) < 85% of median BMI for age and sex, were recruited from consecutive eating disorder admissions at Westmead Hospital, Australia. Weekly measurements of cfPWV were performed for up to 4 consecutive weeks.

**Results:**

12 participants were included, with an average follow-up of 3.2 ± 1.1 weeks. Using mixed-effects models, we observed a significant increase in BMI (95% CI 0.60, 0.80; *p* < 0.01) along with a modest but statistically significant decrease in cfPWV. The rate of change in cfPWV observed was − 0.2 m/s per week (95% CI − 0.37, − 0.03; *p* = 0.03). Mean arterial pressure (MAP) was significantly associated with cfPWV (*p* < 0.01). There was a borderline association between cfPWV and BMI (*p* = 0.05).

**Conclusions:**

Our findings suggest a possible reduction in arterial stiffness with weight restoration, although results must be interpreted with caution due to the small sample. Nevertheless, serial measurements of cfPWV in this population are feasible, supporting the need for larger longitudinal studies in this population.

*Level of evidence*: Level III.

## Introduction

Cardiovascular complications are recognized as a contributing factor to the high mortality rate of anorexia nervosa (AN) [1]. Arterial stiffening is one of the earliest indicators of cardiovascular risk [2]. It reflects the reduced ability of an artery to expand and contract in response to pressure changes. Stiffening of the arteries naturally progresses with age, but the process can be accelerated with certain conditions, such as hypertension and diabetes [3].

Based on recent evidence, adolescents with AN have increased arterial stiffness compared to healthy controls, suggesting heightened risk for future cardiovascular disease [4, 5]. Similar findings have been reported in communities with low body mass index (BMI) [6–8]. The timely recognition of these subclinical vascular changes can slow or prevent progression to cardiovascular disease over the lifespan of patients with AN.

Some cardiovascular changes, including decreased left ventricular mass, bradycardia and heart rate variability can improve with weight restoration in patients with AN [9]. However, there are limited data on the effect of weight restoration on arterial stiffness. Human and animal studies suggest that arterial stiffness may be reversible under specific conditions [10].

While various methods are available to assess arterial stiffness, carotid–femoral pulse wave velocity (cfPWV) is considered the non-invasive gold standard for central arterial stiffness measurement [11]. Higher cfPWV indicates stiffer arteries and has been shown to independently predict cardiovascular events and all-cause mortality across patient groups and in the general population [12, 13].

The underlying mechanisms for the vascular changes in AN remain unclear. Across studies, there has not been a consistent correlation between arterial stiffness and BMI, triglycerides, cholesterol, heart rate, or blood pressure in patients with AN. On the other hand, arterial stiffness has been found to be associated with psychological distress in patients with AN [5]. There is a high rate of psychiatric comorbidities in AN, including mood and anxiety disorders [14]. The role of mental health in the development of cardiovascular diseases is increasingly acknowledged [15].

## Aims

The objective of the study was to assess the change in arterial stiffness during weight restoration in adolescents with AN admitted to an eating disorder unit.

As a secondary aim, we explored the role of psychological distress on arterial stiffness.

This was a pilot study to establish the feasibility of serial cfPWV measurements in our population and to generate information for sample size calculations for future longitudinal studies.

## Methodology

This was a single-center prospective longitudinal study performed at Westmead Hospital, Australia.

### Participants

From January to November 2023, 12 female participants aged between 15 and 19 years were enrolled from consecutive eating disorder admissions to the inpatient eating disorder services at Westmead Hospital. Participants were confirmed to have a diagnosis of AN, made clinically by a multidisciplinary team, including adolescent medicine and mental health clinicians, according to the Diagnostic and Statistical Manual of Mental Disorders, Fifth Edition, Text Revision (DSM-5 TR) and a BMI < 85% of median BMI for age and sex.

Patients with known cardiovascular disease, diabetes, pre-existing renal disease, hypertension, dyslipidaemia, psychosis, or mania were excluded. Patients who were pregnant or breastfeeding, or who had a history of smoking, vaping or substance use were also excluded from the study.

Written informed consent was provided by all participants, and by parents if the participants were less than 18 years. The study was approved by the Sydney Children’s Hospital Network (SCHN) Human Research Ethics Committee (HREC), Australia.

### Data collection

cfPWV measurements and anthropometrics were assessed weekly for up to 4 consecutive weeks during the hospital admission. The first measurement session occurred within the first 5 days of admission after medical stabilization and the initiation of refeeding. The eating disorder unit at Westmead Hospital has an established protocol of using a more rapid approach to refeeding in AN patients. Patients were typically refed commencing with continuous nasogastric feeds for 24–72 h, followed by nocturnal nasogastric feeds and a meal plan of 1800–3800 kcal/day once medically stable.

Demographic information was collected from medical history and patients’ medical records. Height and weight parameters were obtained post voiding before breakfast. BMI was calculated based on the following formula: weight (kg)/height (m)^2^. Centres for Disease Control and Prevention (CDC) standardized growth charts were used for calculating weight, height and BMI centiles.

Urine specific gravity was tested using urinalysis strips (Multistix 10 SG, Siemens, NY, USA) and compared with the Multistix color chart. Urinalysis was performed on the participants before weighing. Pubertal status was self-reported using standardized diagrams on breast development based on the basis of the five-stage criteria described by Tanner [16].

Blood pressure (mmHg) was measured using an automated device (Welch Allyn, NY, USA) on the right arm in the sitting position after a 5-min rest just before the cfPWV was measured. Three consecutive blood pressure measurements were taken (separated by 1–2 min) using the appropriate cuff size. The mean value of the 3 measurements of systolic blood pressure (SBP) and diastolic blood pressure (DBP) was recorded [17, 18]. Mean arterial pressure (MAP) was calculated as: DBP + [(1/3) × (SBP–DBP)].

### Carotid–femoral pulse wave velocity (cfPWV)

cfPWV was measured by a single trained and experienced operator using the SphygmoCor (Atcor Medical, Australia) applanation tonometer device. This is a non-invasive and validated device for measurement of cfPWV [19]. It has been used in a multitude of population studies since 2008 and is well-established in studies involving children and adolescents [19, 20].

The cfPWV measurements were performed at the same time of day (morning before breakfast) for all participants. The majority of participants were on overnight continuous nasogastric feeds which were ceased at least 1 h prior to cfPWV measurement. All measurements were performed in a quiet room with stable room temperature, with the participant lying supine for at least 10 min prior to the measurements. The measurements were repeated by the same operator for all participants over the course of the study.

Pressure waveforms were recorded from the right carotid artery and the right femoral artery sequentially a short time apart using a tonometer. The transit time between the two sites was automatically calculated by the device relative to the R-wave within the electrocardiogram (ECG) complex using the ‘foot-to-foot method’ and the intersecting tangent algorithm. The pulse waveform was captured for 10 s by the device.

The travel distance, defined as the distance traveled by the pulse wave, was measured with a tape measure in a straight line, subtracting the carotid–sternal notch distance from the femoral–sternal notch distance. The final calculation of cfPWV in meters per second (m/s) is the travel distance divided by the transit time.

As recommended by standard guidelines, cfPWV was measured twice, and the mean calculated. If the difference in velocity between the two measurements was > 0.5 m/s, a third measurement was collected. The median of the three measurements was then used in the analysis [11, 21]. Based on the latest data from the Youth Vascular Consortium [22], the 50th percentile of the reference values for cfPWV measured using SphymoCor in 17-year-old healthy adolescent females is 5.17 m/s (10th–90th percentile: 4.24–6.35 m/s).

### Secondary outcomes

The Depression Anxiety Stress Scale (DASS-21) is a 21-item self-report scale used to measure the three related negative emotional states of depression (DASS-D), anxiety (DASS-A) and stress (DASS-S) [23]. Higher scores indicate increased distress (range 0–42). The thresholds for depression are 0–9 (normal), 10–13 (mild), 14–20 (moderate), 21–27 (severe), and 28 or higher (extremely severe). For anxiety, the corresponding ranges are 0–7 (normal), 8–9 (mild), 10–14 (moderate), 15–19 (severe), and 20 or above (extremely severe). Stress levels are classified as 0–14 (normal), 15–18 (mild), 19–25 (moderate), 26–33 (severe), and 34 or higher (extremely severe).

The State–Trait Anxiety Inventory (STAI) is a 40-item self-report scale used to measure anxiety [24]. The state anxiety subscale (STAI-S) assesses the current state of anxiety, asking respondents how they feel “right now”, whereas the trait anxiety subscale (STAI-T) evaluates comparably stable aspects of “anxiety proneness”. Both subscales are calculated from 20 items (range 20–80). A cutoff score of 40 is commonly used to define probable clinical levels of anxiety [25].

DASS-21 and STAI were administered at the beginning and end of the study.

### Statistical analysis

Statistical analyses were conducted by a hospital statistician using Stata SE version 14.2 (StataCorp, College Station, TX, USA). Two-sided *p* values of less than 0.05 were considered statistically significant.

Normally distributed continuous variables were presented as means with standard deviations, and changes between timepoints were assessed using paired *t* tests. Non-normally distributed variables were summarized as medians with interquartile ranges. Categorical variables were reported as frequencies and percentages.

To assess how outcome variables changed over 4 weeks, cfPWV, BMI, heart rate, MAP, DASS-D, DASS-A, DASS-S, STAI-S and STAI-T scores were regressed against time using linear mixed-effects models. To examine predictors of cfPWV, univariate models were fitted separately for BMI, heart rate, MAP, DASS-D, DASS-A, DASS-S, STAI-S, STAI-T, and urine specific gravity. Given the small sample size (*n* = 12) and the risk of overfitting, multivariate analyses were restricted to BMI and MAP, selected for their physiological relevance and significant univariate associations with cfPWV.

A random intercept was specified for each participant to account for repeated measurements over time. Linear mixed-effects models accommodate missing data under the assumption of missing at random using all available observations and appropriately adjusting standard errors and confidence intervals. No additional imputation was performed. Time-by-covariate interaction terms were not included given the limited sample size and short follow-up period.

## Results

Table 1 shows the baseline data of our study population (*n* = 12). The mean age was 17.0 ± 0.7 years, with a mean BMI on admission of 15.5 ± 1.3 kg/m^2^. The mean cfPWV at baseline was 5.19 ± 0.92 m/s.

**Table 1:** Baseline characteristics of the study cohort

Variables	Total (*n* = 12)
Age (years)	17.0 ± 0.7
Anorexia nervosa subtype (*n*)	
Restricting type	9 (75.0%)
Binge-eating/purging type	3 (25.0%)
Excessive exercise^a^ (*n*)	8 (66.7%)
Age of AN onset (years)	15.9 ± 0.8
Duration of illness (months)	12.8 ± 9.9
Previous hospitalizations for AN (*n*)	1 (0–2)
Amenorrhea status (*n*)	
No amenorrhea	2 (16.7%)
< 6 months	5 (41.7%)
≥ 6 months	5 (41.7%)
Height (cm)	163.4 ± 9.2
Weight (kg)	41.5 ± 5.7
BMI (kg/m^2^)	15.5 ± 1.3
HR (bpm)	77.8 ± 11.5
SBP (mmHg)	106.7 ± 6.8
DBP (mmHg)	70.3 ± 5.8
MAP (mmHg)	82.4 ± 6.0
cfPWV (m/s)	5.19 ± 0.92
Urine specific gravity	1.010 (1.005–1.014)
TSH (mIU/L)	1.47 ± 0.41
T4 (pmol/L)	11.4 ± 1.2
T3 (pmol/L)	3.3 ± 0.44
FSH (IU/L)	4.5 (0.0–6.0)
LH (IU/L)	0.3 (0.0–1.0)
Oestradiol (pmol/L)	< 100 (0–100)

Data values are shown as means ± standard deviations or median (interquartile range) for continuous variables. Data values are shown in numbers (%) for categorical variables

^a^Excessive exercisers defined as persons with 6 or more episodes of driven exercise within the past 7 days

*AN* anorexia nervosa, *BMI* body mass index, *HR* heart rate, *SBP* systolic blood pressure, *DBP* diastolic blood pressure, *MAP* mean arterial pressure, *cfPWV* carotid–femoral pulse wave velocity, *TSH* thyroid-stimulating hormone, *T4* thyroxine, *T3* triiodothyronine, *FSH* follicle-stimulating hormone, *LH* luteinizing hormone

Eleven of the participants were on one or more psychotropic medications during the study (Table 2). More than half of the participants experienced severe to extremely severe levels of depression, anxiety and stress at baseline. Almost all participants (90.9%) were classified as having meaningful anxiety (STAI score > 40).

**Table 2:** Baseline mental health characteristics

Medications (*n*)	
Antipsychotics	10 (83.3%)
SSRI	5 (41.7%)
Mood stabilizer	2 (16.7%)
DASS depression	28.2 ± 6.6
Normal	0
Mild	0
Moderate	2 (18.2%)
Severe	2 (18.2%)
Extremely severe	7 (63.6%)
DASS anxiety	25.6 ± 8.2
Normal	0
Mild	1 (9.1%)
Moderate	0
Severe	1 (9.1%)
Extremely severe	9 (81.8%)
DASS stress	28.4 ± 6.8
Normal	0
Mild	1 (9.1%)
Moderate	3 (27.3%)
Severe	3 (27.3%)
Extremely severe	4 (36.4%)
STAI-S	57.9 ± 13.6
STAI-T	62.0 ± 12.0

*SSRI* selective serotonin reuptake inhibitor, *DASS* Depression Anxiety Stress Scale, *STAI-S* State–Trait Anxiety Inventory–state anxiety subscale, *STAI-T* State–Trait Anxiety Inventory–trait anxiety subscale

Out of 12 trial participants, 4 were discharged from hospital prior to the 4-week mark. One participant opted out of the study after a week. The average follow-up time was 3.2 ± 1.1 weeks. During the duration of hospital admission, the mean BMI increased from 15.5 ± 1.3 to 18.1 ± 1.7 kg/m^2^ (*p* < 0.001). An average weight gain of 7.2% was achieved among the participants.

**Fig. 1:**
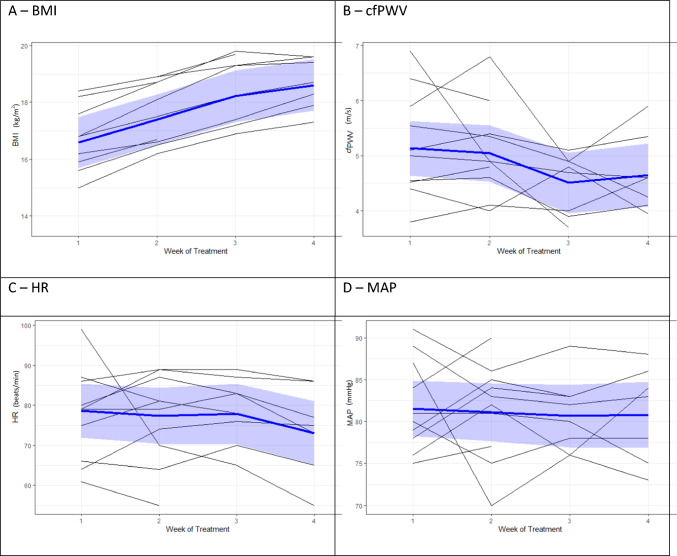
Graphs of changes in key variables over time

Figure 1 shows the graphs of changes in key variables over time during the study. As BMI increased, cfPWV showed a decreasing trend. Heart rate and mean arterial pressure remained fairly stable.

We investigated the trend above further using the linear mixed-effects model (Table 3). In parallel with the increase in BMI (95% CI 0.60, 0.80; *p* < 0.01), there was a smaller but significant decrease in cfPWV. The rate of change in cfPWV observed was − 0.2 m/s per week (95% CI − 0.37, − 0.03; *p* = 0.03), corresponding to an estimated marginal mean of 4.5 m/s (95% CI 4.0–5.1) at week 4. There were no significant changes with HR and MAP over time. The DASS and STAI scores decreased over time, but none of the differences were statistically significant (*p* > 0.05).

**Table 3:** Linear mixed-effects models examining the change in variables over time

Variable	Coefficient (95% CIs)	*p* value
BMI	0.70 (0.60, 0.80)	< 0.01
cfPWV	− 0.20 (-0.37, -0.03)	0.03
HR	− 1.53 (-3.87, 0.87)	0.21
MAP	− 0.29 (-1.50, 0.94)	0.64
DASS depression	− 0.57 (-2.42, 1.26)	0.54
DASS anxiety	− 1.32 (-3.25, -0.61)	0.24
DASS stress	− 1.34 (-2.84, 0.16)	0.12
STAI-S	− 1.08 (-4.92, 2.77)	0.87
STAI-T	− 0.16(-2.06, 1.74)	0.60

*BMI* body mass index, *cfPWV* carotid–femoral pulse wave velocity, *HR* heart rate, *MAP* mean arterial pressure, *DASS* Depression Anxiety Stress Scale, *STAI-S* State–Trait Anxiety Inventory-state anxiety subscale, *STAI-T* State–Trait Anxiety Inventory-trait anxiety subscale

As shown in Table 4, MAP was significantly associated with cfPWV (*p* < 0.01). There was a borderline association between cfPWV and BMI (*p* = 0.05). We subsequently ran an additional multivariate model to investigate the relationships between cfPWV, BMI and MAP. BMI was borderline significant (*p* = 0.07) with a similar size coefficient to that of the univariate model (β − 0.15; 95% CI − 0.30, 0.01), while MAP was significant (*p* < 0.01) with a similar size coefficient to that of the univariate model (β 0.09; 95% CI 0.05, 0.14). Albeit limited by the small sample size, it is likely that both BMI and MAP have independent relationships with cfPWV. The DASS and STAI scores were not associated with changes in cfPWV.

**Table 4:** Linear mixed-effects models examining the association between cfPWV and variables

Variable	Coefficient (95% CIs)	*p* value
BMI	− 0.19 (-0.40, 0.04)	0.05
HR	0.02 (-0.01, 0.05)	0.08
MAP	0.09 (0.05, 0.13)	< 0.01
SG	− 5.99 (-52.5, 39.4)	0.80
DASS depression	0.0038 (-0.059, 0.066)	0.91
DASS anxiety	0.024 (-0.027, 0.076)	0.37
DASS stress	− 0.026 (-0.079, 0.028)	0.37
STAI-S	− 0.0037(-0.032, 0.024)	0.8
STAI-T	− 0.015(-0.058, 0.028)	0.50

Linear mixed-effects models of individual repeated measures of BMI, HR, MAP, SG, DASS and STAI as predictors of cfPWV

*BMI* body mass index, *HR* heart rate, *MAP* mean arterial pressure, *SG* urine specific gravity, *DASS* Depression Anxiety and Stress Scale, *STAI-S* State–Trait Anxiety Inventory-state anxiety subscale, *STAI-T* State–Trait Anxiety Inventory–trait anxiety subscale

## Discussion

This pilot study supports the feasibility of serial cfPWV measurements in adolescents with AN and provides preliminary data on early vascular changes during refeeding. Although baseline cfPWV values were within the published reference ranges for healthy adolescents and may not be clinically concerning in isolation [22], we observed a reduction in arterial stiffness during the first 4 weeks of nutritional rehabilitation. BMI increased by 0.7 kg/m^2^ a week with a corresponding decrease in cfPWV of 0.2 m/s a week. While the clinical significance of this change remains uncertain, the findings suggest that some vascular effects of malnutrition may be at least partially reversible with weight restoration.

The observed weekly decline in cfPWV was greater than that reported by Hudson et al. (− 0.20 m/s per week vs. − 0.05 m/s per week), but this effect may be inflated given the small pilot cohort [26]. Using a more conservative estimated effect size of 0.05 m/s per week, calculations suggest that a sample of 28 participants will be required to achieve a power of 0.8 for a future longitudinal study of this type, assuming a significance level of 0.05.

While there was a modest association between BMI and PWV, blood pressure was a strong predictor of PWV. However, given the small sample size, the associations between BMI, blood pressure, and cfPWV should, therefore, be considered exploratory.

Our results add to a growing but heterogeneous literature on vascular health in AN. Cross-sectional studies have consistently shown increased arterial stiffness in adolescents and young adults with AN compared with healthy controls [4, 5, 27], with some evidence of persistence even after weight restoration or into adulthood [5, 28]. For example, Springall et al. reported increased carotid stiffness, but decreased aortic stiffness, alongside endothelial dysfunction in young adults with prior AN, suggesting potential long-term sequelae [28]. Our observation of reduced arterial stiffness during early refeeding aligns with Hudson et al. [26], supporting the hypothesis that some vascular changes in AN may be functional and reversible, but others, such as structural or endothelial abnormalities may persist beyond recovery. Taken together, these studies highlight the complexity of vascular adaptation in AN.

The mechanisms underlying altered arterial stiffness in AN are likely multifactorial. Low BMI may increase arterial stiffness through enhanced oxidative stress, reduced bioavailability of nitric oxide, and endothelial dysfunction, leading to accelerated vascular aging and arteriosclerosis [5, 29]. Structural vascular alterations, autonomic dysfunction (altered symphathetic and parasympathetic tone) and hormonal factors, such as oestrogen deficiency, a known consequence of AN, may also affect arterial compliance [28, 30–32].

In contrast to Jenkins et al. [5], we did not observe associations between arterial stiffness and psychological distress. This may reflect limited statistical power and the relatively short study duration. Furthermore, almost all participants reported high baseline levels of distress, resulting in limited variability in scores during hospital admission, which reduces the ability to detect correlations with arterial stiffness.

## Strengths and limits

The strengths of the study include its longitudinal design during a critical phase of nutritional rehabilitation and the homogeneity of the cfPWV measurements and technique. All measurements were performed by a single operator using SphygmoCor, adhering to the latest recommendations for cfPWV measurement [11, 21]. The methodology was well-tolerated by participants with unproblematic recruitment. While we did not study a healthy control group for comparison, we were able to compare our baseline distribution of cfPWV to published data in healthy populations measured using a similar device [22].

This study has limitations that should be considered when interpreting the results. First, the data are based on a relatively small sample of exclusively female adolescents with AN admitted to a single inpatient eating disorder unit. It may not be possible to extrapolate the findings to males or other stages of illness. We had a comparatively short follow-up time of 4 weeks, which was approximately the average length of stay for patients admitted to our eating disorder unit. Larger studies with longer follow-up periods are needed to clarify these trajectories. The absence of direct measures of autonomic function or endothelial biomarkers precludes interpretation about underlying mechanisms.

Almost all participants were prescribed psychotropic medications, particularly second-generation antipsychotics and SSRIs, which may confound vascular outcomes, although evidence of their effect on PWV in adolescents is limited [33–36]. As formal assessments by psychological professionals were not feasible during this study, the use of non-diagnostic self-report questionnaires as proxies for depression and anxiety is subject to measurement and reporting bias.

## Conclusion

Measurement of cfPWV is non-invasive and highly reproducible, making it feasible for larger longitudinal studies in adolescents with anorexia nervosa. This pilot study suggests that arterial stiffness may improve with weight restoration, though findings must be interpreted with caution due to the small sample size. The complexity of the underlying mechanisms behind cfPWV changes in anorexia nervosa warrants further investigation.

## Summary table

**Table:** 

What is known about topic?	What this study adds?
• Pulse wave velocity is a marker of arterial stiffness and early cardiovascular risk	• Adolescent focus
• Adolescents with anorexia nervosa show increased arterial stiffness compared to healthy controls	• Weekly measurements of pulse wave velocity in adolescents with anorexia nervosa were feasible in our cohort
• The impact of weight restoration on arterial stiffness in anorexia nervosa remains unclear	• In this small sample, pulse wave velocity decreased after 4 weeks of inpatient refeeding, as BMI increased

## Data Availability

The data sets generated during and/or analyzed during the current study are available from the corresponding author on reasonable request.
